# The longitudinal relationship between dissociative symptoms and self-harm in adolescents: a population-based cohort study

**DOI:** 10.1007/s00787-023-02183-y

**Published:** 2023-03-08

**Authors:** Riki Tanaka, Shuntaro Ando, Tomoki Kiyono, Rin Minami, Kaori Endo, Mitsuhiro Miyashita, Syudo Yamasaki, Sho Kanata, Shinya Fujikawa, Mariko Hiraiwa-Hasegawa, Atsushi Nishida, Kiyoto Kasai

**Affiliations:** 1 Department of Neuropsychiatry, Graduate School of Medicine, The University of Tokyo, 7-3-1 Hongo, Bunkyo-Ku, Tokyo, Japan; 2 Department of Psychiatry, Teikyo University Mizonokuchi Hospital, Kanagawa, Japan; 3 Research Center for Social Science and Medicine, Tokyo Metropolitan Institute of Medical Science, Tokyo, Japan; 4 Department of Psychiatry, Teikyo University School of Medicine, Tokyo, Japan; 5 School of Advanced Science, SOKENDAI (Graduate University for Advanced Studies), Kanagawa, Japan

**Keywords:** Dissociation, Self-injurious behavior, Adolescent, Longitudinal studies, Birth cohort

## Abstract

Previous studies have reported that dissociative symptoms (DIS) are associated with self-harm (SH) in adolescents. However, most of these studies were cross-sectional, which limits the understanding of their theoretical relationship. We aimed to investigate the longitudinal relationship between DIS and SH in the general adolescent population. We used data from the Tokyo Teen Cohort study (*N* = 3007). DIS and SH were assessed at times 1 and 2 (T1 and T2) (12 years of age and 14 years of age, respectively). DIS were assessed using the parent-report Child Behavior Checklist (CBCL), and severe dissociative symptoms (SDIS) were defined as a score above the top 10th percentile. The experience of SH within 1 year was assessed by a self-report questionnaire. The longitudinal relationship between DIS and SH was examined using regression analyses. Using logistic regression analyses, we further investigated the risk for SH at T2 due to persistent SDIS and vice versa. DIS at T1 tended to predict SH at T2 (odds ratio (OR) 1.11, 95% CI 0.99 to 1.25, *p* = 0.08), while SH at T1 did not predict DIS at T2 (B = − 0.03, 95% CI − 0.26 to 0.20, *p* = 0.81). Compared with adolescents without SDIS, those with persistent SDIS had an increased risk of SH at T2 (OR 2.61, 95% CI 1.28 to 5.33, *p* = 0.01). DIS tended to predict future SH, but SH did not predict future DIS. DIS may be a target to prevent SH in adolescents. Intensive attention should be given to adolescents with SDIS due to their increased risk of SH.

**Supplementary Information:**

The online version contains supplementary material available at 10.1007/s00787-023-02183-y.

## Introduction

Self-harm (SH) is one of the major public health problems in adolescents, and approximately 10% of adolescents in the community engage in SH. [1–4] While the majority of adolescents stop SH in young adulthood, SH as a maladaptive coping behavior may continue into young adulthood [3]. A study with a cohort of young patients presenting to hospitals after SH revealed that the risks of death and suicide were four times and ten times greater than expected, respectively [5]. Therefore, it is important to investigate the risks of SH and the mechanisms by which it is initiated and maintained.

A number of cross-sectional studies have suggested an association between dissociative symptoms (DIS) and SH in both community [6–11] and clinical [12–15] populations of adolescents. DIS refers to a deficit in the continuity of subjective experiences such as depersonalization, derealization or the inability to control one’s mental functions [16]. DIS are a public health problem not only in the clinical population but also in the general population. [17] DIS are closely related to childhood trauma and abuse from family members. [18][18] For the relationship between DIS and SH, temporally conflicting hypotheses have been argued. According to a previous study, DIS precede SH, and SH may be a strategy to regain a sense of reality and end an unpleasant dissociated state. [20] It has also been suggested that in a severely dissociated person, when one dissociated part becomes aggressive to another, SH could occur. [21] On the other hand, SH may be a trigger to dissociate and escape from unbearable distress [22], and recent neuroimaging findings support this theory. [23, 24] If DIS antecede SH, or vice versa, the repetition of one may increase or worsen the other over a period of time. However, since most studies on the association between DIS and SH were cross-sectional, the longitudinal relationship between them has been unclear [25]. Although a previous study showed that a decrease in DIS significantly predicted a decrease in SH at 9-month intervals in adolescent females who experienced sexual abuse, the sample size was small, and the follow-up rate was low [26]. To date, no study has examined the longitudinal relationship between DIS and SH in the general population of adolescents.

A community-based longitudinal study is required for the development of a generalizable prevention strategy because previous studies have revealed that only a small portion of young people receive medical treatment for SH [1]. Therefore, we aimed to investigate the longitudinal relationship between DIS and SH using data from a cohort study of the general adolescent population. Specifically, we aimed to examine the two-way longitudinal relationship between DIS and SH. Furthermore, we aimed to examine the risk of SH due to persistent severe dissociative symptoms (SDIS) and vice versa.

## Methods

### Data and samples

Our data were derived from the Tokyo Teen Cohort (TTC) study, which is an ongoing longitudinal birth cohort study of adolescents who were born between September 2002 and August 2004. The study participants were randomly sampled using the resident resister in three neighboring municipalities in Tokyo: Setagaya-ku, Mitaka-shi, and Chofu-shi. The details of the cohort are described elsewhere [27]. This study used data collected when the participants were 12 (time 1; T1) and 14 years of age (time 2; T2). A total of 3007 pairs of adolescents and their parents participated in the T1 survey, and 2667 pairs participated in the T2 survey. For each survey, home visits were conducted twice for data collection. At the first visit, written informed consent was obtained from the parents. The adolescents and their parents were asked to complete the questionnaires at home before the second visit. At the second visit, adolescents and their parents were asked to complete the self-report questionnaires separately and enclose the questionnaires in the envelopes by themselves immediately after completion. The TTC study is supported by three research institutes: the Tokyo Metropolitan Institute of Medical Science (approval number: 12–35), the University of Tokyo (approval number: 10057), and SOKENDAI (The Graduate University for Advanced Studies) (approval number: 2012002). This study was approved by the ethics committees of the three institutes mentioned above.

### Measures

#### Dissociative symptoms

DIS refer to unwanted intrusions into awareness and behavior, with accompanying deficits in the continuity of subjective experiences (e.g., identity fragmentation, depersonalization, derealization) or the inability to access information or control mental functions (e.g., amnesia, aphonia, paralysis) [16]. As in previous studies [28, 29], DIS were assessed by a parent-report questionnaire using six items from the Child Behavior Checklist, a widely used questionnaire for identifying problematic behavior in children [30]. Since the ability of meta-cognition changes dramatically in adolescence [31, 32], we considered that ratings by parents were more stable than ratings by children. We adopted CBCL assessment, while previous cross-sectional studies on the relationship of DIS and SH adopted self-report assessment [6–11]. The six items were selected because they were similar to the items of the Child Dissociative Checklist (CDC), a standard measure of DIS in children [33]. They showed reasonable internal consistency (Cronbach’s alpha: 0.71). [34] Assessment of DIS by the CBCL was shown to positively correlate with the CDC in abused and non-abused children (r = 0.63). [34] These items were also used in previous studies with participants in a relatively close age group [28, 29] and demonstrated acceptable internal consistency (Cronbach’s alpha: 0.57 to 0.68). [28, 34]Specifically, we used the following items: i) “acts too young for his or her age,” ii) “cannot concentrate, cannot pay attention for a long period of time,” iii) “confused or seems to be in a fog,” iv) “daydreams or gets lost in his or her thoughts,” v) “stares blankly,” and vi) “sudden changes in mood or feelings.” The parent answered on a three-point scale: not true = 0, somewhat or sometimes true = 1, and very true or often true = 2. The DIS score was defined as the sum of the answers to these six questions (possible range: 0–12). The Cronbach’s alpha of these items was 0.61 at T1 and 0.68 at T2. The prevalence of dissociative disorders in the general population is reported to range from 1.7% to 18.3% [17], and the prevalence of a pathological level of dissociation was reported to be approximately 15% in Japanese adolescents in the community [35]. Since a previous study defined the top 10th percentile of Adolescent Dissociative Experiences Scale (ADES) scores as pathological dissociation [7], we defined severe DIS (SDIS) as a DIS score above the top 10th percentile. In this study, the cutoff DIS score for SDIS was set at 5 at both T1 and T2. To eliminate arbitrariness and to confirm the stability of the results, we conducted sub-analyses using different cutoffs of SDIS, specifically the top 5th percentile for narrow SDIS and the top 20th percentile for broad SDIS. The cutoff DIS scores were 6 and 4, respectively.

### Self-harm

SH refers to intentional self-injury or self-poisoning regardless of suicidal intent [36]. SH was assessed by a self-report questionnaire. At T1, the adolescents answered the following question with a response of yes or no: “Have you ever intentionally hurt yourself in the past year?”. At T2, they answered the same question with the following response options: (1) never, (2) only once, (3) 2 to 5 times, (4) 6 to 10 times, and (5) more than 10 times. They were also asked to give free descriptions to the following questions: “Please list any body parts that have been injured within the past year” and “Please describe the ways in which you have hurt yourself within the past year”. Four psychiatrists (SA, TK, RM, and RT) determined whether the responses could be considered SH by referring to the definition of non-suicidal self-injury presented by the International Society for the Study of Self-Injury (https://www.itriples.org/). SH was defined as meeting the following requirements: 1) the harm was intentional or expected, 2) SH usually resulted in immediate physical injury, and 3) socially sanctioned injuries were not considered SH. In this study, we did not take SH motivations into account. Thus, we coded 1 when the adolescents had ever intentionally hurt themselves in the past year and 0 when they had never intentionally hurt themselves.

### Statistical analysis

Regression analyses were conducted to examine whether SH at T1 predicted DIS at T2. We adjusted for age, sex, and DIS at T1. Multiple logistic regression analyses were conducted to examine whether DIS at T1 predicted SH at T2. We adjusted for age, sex, and SH at T1. The interaction terms DIS at T1 × sex and SH at T1 × sex were also added to each adjusted model to examine whether there were sex-related interactions. Subsequently, we divided the participants into four groups depending on the trajectory of SDIS over the 2 years: the no experience group (T1 (−), T2 (−)), incident group (T1 (−), T2 (+)), transient group (T1 (+), T2 (−)), and persistent group (T1 (+), T2 (+)). The risk of SH in each group at T2 was investigated by logistic regression analysis with the no experience group as a reference. For the trajectory of SH, we also divided the participants into four groups in the same way: no experience group (T1 (−), T2 (−)), incident group (T1 (−), T2 (+)), transient group (T1 (+), T2 (−)), and persistent group (T1 (+), T2 (+)). We investigated the risk of SDIS at T2 depending on the trajectory of SH over the 2 years in the same way. In these analyses, age and sex were also included as covariates. We applied a multiple imputation method to handle missing values under the assumption of missing at random. In the imputation models, we included all variables used in the analysis along with several additional auxiliary variables. Additional auxiliary variables were obtained by the prior data collection survey at 10 years of age. The included variables were as follows: DIS at T1, DIS at T2, SH at T1, SH at T2, sex, age in months at T1, age in months at T2, the interaction term DIS at T1 × sex, the interaction term SH at T1 × sex, IQ score by the Wechsler Intelligence Scale for Children—Fourth Edition[37], the father’s educational history, the mother’s educational history, the mother’s estimated IQ score by the Japanese version of the National Adult Reading Test[38], the mother’s age, and annual household income. By fully conditional specification, 100 imputed datasets were generated, and the analyses were conducted for these datasets. As sub-analyses, pairwise deletion was conducted excluding participants who had missing data from the analysis. The significance level was set at 0.05. IBM^®^ SPSS^®^ Statistics version 28.0 (IBM^®^ Corp., Armonk, NY, USA) was used for statistical analysis.

## Results

### Demographic characteristics of the participants

The female percentage of the participants was 47.0% at T1 and 48.2% at T2 (Table 1). All participants were Asian. For the DIS score, the minimum score was 0, the maximum score was 11, and the median score was 2 at both T1 and T2. The cross-sectional correlations between DIS and SH were significant at both T1 (Spearman’s rank correlation coefficient (*rs* = 0.088, 95% CI 0.048 to 0.128, *p* < 0.001) and T2 (*rs* = 0.086, 95% CI 0.041 to 0.130, p < 0.001). Of the 3007 total participants, the number of respondents for each variable ranged from 2023 (SH at T2) to 3007 (sex).

**Table 1:** Demographic characteristics of the study participants (*n* = 3007, female: 47%)

	T1				T2			
	Total	Male	Female	*p* value^1^	Total	Male	Female	*p* value^1^
Age in months, mean (SD)	146.03 (3.66)	146.03	146.03	0.99	172.41 (3.44)	172.46	172.34	0.37
DIS score, mean (SD)	1.98 (1.78)	2.15	1.79	< 0.01	2.00 (1.94)	2.12	1.86	< 0.01
SH, %	11.2	11.0	11.4	0.77	4.3	2.4	6.4	< 0.01

T1: 12 years of age, T2: 14 years of age, *DIS score*: CBCL scores indicating dissociative symptoms, *SH* self-harm, ^1^*p* value for sex differences

### Longitudinal relationship between DIS and SH

As a result of regression analyses using the multiple imputation method, after controlling for age, sex, and SH at T1, DIS at T1 tended to predict SH at T2 (odds ratio (OR) 1.11, 95% CI 0.99 to 1.25, *p* = 0.08) (Table 2). There was no significant interaction between DIS and sex (*p* = 0.33). The sub-analysis using the pairwise deletion method showed that DIS at T1 predicted SH at T2 (odds ratio (OR) 1.14, 95% CI 1.01 to 1.28, *p* = 0.03) (Table 2).

**Table 2:** Multiple logistic regression analysis of the effects of dissociative symptoms at T1 on self-harm at T2

	Unadjusted			Adjusted^1^		
	OR	95% CI	*p* value	OR	95% CI	*p* value
DIS at T1, with multiple imputation	1.10	0.98 to 1.24	0.11	1.11	0.99 to 1.25	0.08
DIS at T1, with pairwise deletion	1.11	0.99 to 1.23	0.08	1.14	1.01 to 1.28	0.03

T1: 12 years of age, T2: 14 years of age, *DIS* CBCL scores indicating dissociative symptoms

^1^adjusted for self-harm at 12 years of age, sex and age in months

On the other hand, regression analyses using the multiple imputation method showed that after controlling for age, sex, and DIS at T1, SH at T1 did not predict DIS at T2 (*B* = − 0.03, 95% CI − 0.26 to 0.20, *p* = 0.81) (Table 3). There was no significant interaction between SH and sex (*p* = 0.95). The sub-analysis using the pairwise deletion method also showed that SH at T1 did not predict DIS at T2 (B = − 0.01, 95% CI − 0.22 to 0.20, *p* = 0.92) (Table 3).

**Table 3:** Multiple regression analysis of the effects of self-harm at T1 on dissociative symptoms at T2

	Unadjusted			Adjusted^1^		
	*B*	95% CI	*p* value	*B*	95% CI	*p* value
SH at T1, with multiple imputation	0.26	− 0.10 to 0.62	0.16	− 0.03	− 0.26 to 0.20	0.81
SH at T1, with pairwise deletion	0.39	0.12 to 0.66	< 0.01	− 0.01	− 0.22 to 0.20	0.92

T1: 12 years of age, T2: 14 years of age, *SH* self-harm

^1^adjusted for dissociative symptoms at 12 years of age, sex and age in months

### Risk of SH due to the trajectory of SDIS and vice versa

Since we defined SDIS as DIS scores above the top 10th percentile, the cutoff DIS score was set at 5 at both T1 and T2, with a prevalence of SDIS of 9.2% at T1 and 11.0% at T2. After controlling for age and sex, those with persistent SDIS had a significantly increased risk of SH at T2 compared with the no experience group (OR 2.61, 95% CI 1.28 to 5.33, *p* = 0.01) (Table 4). Neither the incident group nor the transient group had an increased risk of SH at T2 (incident group: OR 1.76, 95% CI 0.81 to 3.81, *p* = 0.15; transient group: OR 0.80, 95% CI 0.21 to 3.03, *p* = 0.74) (Table 4). When we defined narrow SDIS as above the top 5th percentile, the cutoff DIS score was 6 at both T1 and T2, with a prevalence of narrow SDIS of 4.8% at T1 and 5.9% at T2. After controlling for age and sex, those with persistent narrow SDIS had a significantly increased risk of SH at T2 compared with the no experience group (OR 4.31, 95% CI 1.87 to 9.94, *p* < 0.001) (Supplementary Table 1). When we defined broad SDIS as above the top 20th percentile, the cutoff DIS score was 4 at both T1 and T2, with a prevalence of broad SDIS of 18.1% at T1 and 19.4% at T2. After controlling for age and sex, those with persistent broad SDIS had a significantly increased risk of SH at T2 compared with the no experience group (OR 2.18, 95% CI 1.22 to 3.88, *p* < 0.01) (Supplementary Table 2).

**Table 4:** The trajectory of severe dissociative symptoms and the risk of self-harm

	SDIS at T1	SDIS at T2	Prevalence, %	OR	95% CI	*p* value
Trajectory of SDIS						
No experience	−	−	84.9	1	Reference	
Incident	−	+	5.9	1.76	0.81 to 3.81	0.15
Transient	+	−	4.4	0.80	0.21 to 3.03	0.74
Persistent	+	+	4.9	2.61	1.28 to 5.33	0.01

Adjusted for sex and age in months

*SDIS* severe dissociative symptoms, T1: 12 years of age, T2: 14 years of age

After controlling for age and sex, the persistent SH group tended to be associated with SDIS at T2 (OR 2.30, 95% CI 0.90 to 5.91, *p* = 0.08) compared with the no experience group. The incident SH group was significantly associated with SDIS at T2 (OR 2.14, 95% CI 1.08 to 4.25, *p* = 0.03), while the transient SH group was not (OR 1.23, 95% CI 0.77 to 1.96, *p* = 0.38) (Table 5).

**Table 5:** The trajectory of self-harm and the risk of severe dissociative symptoms

	SH at T1	SH at T2	Prevalence, %	OR	95% CI	*P* value
Trajectory of SH						
No experience	−	−	85.5	1	Reference	
Incident	−	+	3.1	2.14	1.08 to 4.25	0.03
Transient	+	−	10.1	1.23	0.77 to 1.96	0.38
Persistent	+	+	1.3	2.30	0.90 to 5.91	0.08

Adjusted for sex and age in months

*SH* self-harm, T1: 12 years of age, T2: 14 years of age

## Discussion

This was the first study that examined a longitudinal relationship between DIS and SH in the general adolescent population. While DIS tended to predict future SH, SH did not predict future DIS. Individuals with persistent SDIS had an approximately three times higher risk of SH than those with no experience of SDIS. Individuals with persistent SH tended to have a higher risk of SDIS.

The results of this study showed that DIS tended to longitudinally predict future SH. This result is in line with a previous study that showed that a decrease in DIS predicted a decrease in SH in adolescent female victims of sexual abuse [26]. Furthermore, regarding the temporal relationship, the results also agree with previous cross-sectional studies that showed that DIS mediated the relationship between childhood trauma and SH [8, 11, 14, 15, 24] Regarding the empirical and theoretical hypotheses, this longitudinal relationship may support the anti-dissociation model of SH [20]. In previous studies, approximately half of adolescents with SH endorsed the reasons that were assumed to end DIS, such as “to stop feeling numb or out of touch with reality” or “to feel something even if it is pain” [39, 40] A previous study suggested that feeling pain or seeing their blood may be instrumental in retaining reality or a coherent sense of existence and ending DIS. [41] Another explanation is that this longitudinal relationship may also support the self-punishment model of SH. [20] A previous study suggested that SH may be triggered by intrapsychic conflict in severely dissociated persons, in which one dissociated part could be aggressive to another. [21] In both hypotheses, temporal relief after SH as anti-dissociation or self-punishment may reinforce SH and lead to its repetition. [42]

We also revealed that those with persistent SDIS had a significantly increased risk of SH at T2. This suggests that the repetition of SDIS over a 2-year period may lead to the initiation or maintenance of SH. To the best of our knowledge, no study has examined the association between persistent SDIS and SH in the general population of adolescents. While a previous study suggested that the severity and frequency of DIS are associated with the severity of SH, [25, 26] attention should also be given to the persistence of SDIS as a risk factor for SH. Since the transient SDIS group did not have an increased risk for SH, spontaneous transient SDIS may not increase the risk for SH. This result may also suggest the importance of early intervention for preventing future SH in adolescents with SDIS [43].

This study revealed that SH did not predict DIS 2 years later. Although a previous study suggested that SH is a deliberate attempt to dissociate and escape from unbearable distress, [22] SH may not promote a long-lasting tendency to dissociate. Nonetheless, it should be noted that adolescents with persistent SH tended to have an increased risk of SDIS. This is in line with previous findings that habitual SH was associated with DIS [14, 44] although the associations were stronger in previous studies, probably due to the cross-sectional design. This study also revealed that adolescents with incident SH had a significantly higher risk of SDIS. Therapists treating adolescents with SH should pay attention to the comorbidity of SDIS, especially if the SH is persistent or relatively recently initiated.

Although the cross-sectional association between DIS and SH was significant, the longitudinal relationship between DIS and SH was weak or non-significant. The observed significant cross-sectional association between DIS and SH fits with previous findings [6–15, 26]. There may be several explanations for the relationship between DIS and SH that was observed in this study. First, DIS and SH may have a longitudinal relationship over a relatively short period. Since we assessed DIS and SH twice over a relatively long period (2 years), the longitudinal relationship between them might be observed to be weaker. Second, DIS and SH may mostly co-occur rather than one leading to the other. DIS and SH may both occur in adolescents who suffer from acute strong distress. In addition, they may also occur in adolescents who suffer from strong distress when recalling past adverse experiences. For example, childhood trauma is a risk factor for both DIS and SH [11, 14, 15, 36], and a previous study showed that the majority of dissociative disorder patients had self-harmed after trauma-related cues [45].

Several clinical implications can be drawn from this study. First, interventions for DIS should be considered to prevent future SH in adolescents, even if they do not currently engage in SH. The interventions may focus on distress, which promotes DIS. [46] Second, intensive attention should be given to adolescents who have repeated SDIS since they are at a specifically higher risk of SH. Persistent SDIS may be regarded as a representation of unresolved persistent distress.

The strengths of this study include the design of the TTC study. First, this study was longitudinal with a prospective design. Since most previous studies were cross-sectional, the results of our study may contribute to the understanding of the theoretical relationship between DIS and SH. Second, the sample size of this study was relatively large compared to previous cross-sectional studies with general populations [6–10]. Third, this study used a sample of adolescents from the general population. Since only a small portion of adolescents receive medical treatment for SH, [1] this study may contribute to the development of a generalizable prevention strategy.

However, there are some possible limitations. First, we assessed DIS by the parent-report CBCL. There may be concerns about the validity of a parent’s assessment of a child’s internal experience. The parent’s assessment might underestimate or overestimate DIS. Only DIS with more than a substantial severity might be captured by parents, but mild DIS might be missed and underreported. On the other hand, contemplation or meditation might be regarded as DIS by parents and could be over-reported. Since most previous cross-sectional studies on the relationship between DIS and SH used self-report assessment of DIS [6–11], our results should be compared with previous studies carefully. Second, since we assessed SH only by children’s self-reports, the possibility of underreporting should be considered; however, a previous study suggested that self-reports were suitable for highly sensitive questions [47]. In addition, we used one generic question to assess SH at T1, although we assessed SH at T2 in more detail using free description. Although the measurement method was similar to that of previous studies[2, 3, 48, 49], it is a limitation that we did not use validated questionnaires to assess SH. Third, most of the participants were Japanese adolescents living in Tokyo, an Asian metropolis. Careful consideration is needed to generalize these study findings to other ethnic groups and countries. Fourth, we did not obtain information about childhood trauma and abuse from family members because asking about such experience was considered possibly invasive for the adolescents. This may limit the interpretation of the results of this study in conjunction with other previous works. Both DIS and SH were suggested to be associated with childhood trauma [11, 14, 36], while a previous study revealed that a higher level of DIS was related to SH even after controlling for childhood abuse [50]. Further study is needed to examine the longitudinal relationship among DIS, SH, and childhood trauma. Future studies are also warranted to investigate the longitudinal relationship between DIS and SH over a shorter period.

## Conclusion

DIS tended to predict future SH, but SH did not predict future DIS. Adolescents with persistent SDIS had an approximately three times higher risk of SH. Adolescents with persistent SH tended to have a higher risk of SDIS. DIS may be a target to prevent future SH in adolescents. Intensive attention should be given to adolescents with persistent SDIS since they have a rather high risk of SH.

### Supplementary Information

Below is the link to the electronic supplementary material.

Supplementary file1 (DOCX 19 KB)

## Data Availability

The raw data supporting the conclusions of this article will be made available by the authors, without undue reservation.
