# Supplementary material for: The longitudinal relationship between dissociative symptoms and self-harm in adolescents: a population-based cohort study
Source: Eur Child Adolesc Psychiatry. 2023 Mar 8;33(2):561–8. doi: 10.1007/s00787-023-02183-y (PMC10869437; doi:10.1007/s00787-023-02183-y)
Supplement: Supplementary file 1 — Supplementary file1 (DOCX 19 KB) [file 787_2023_2183_MOESM1_ESM.docx]

| Supplementary Table 1: The trajectory of narrow severe dissociative symptoms and the risk of self-harm | | | | | | |
| --- | --- | --- | --- | --- | --- | --- |
|  | narrow SDIS at T1 | narrow SDIS at T2 | prevalence, % | OR | 95% CI | P value |
| Trajectory of narrow SDIS |  |  |  |  |  |  |
| No experience | - | - | 91.5 | 1 | reference |  |
| Incident | - | + | 3.6 | 2.40 | 1.03 to 5.58 | 0.04 |
| Transient | + | - | 2.5 | 0.67 | 0.11 to 4.25 | 0.67 |
| Persistent | + | + | 2.3 | 4.31 | 1.87 to 9.94 | <0.001 |
| Adjusted for sex and age in months. | | | | | | |
| SDIS: severe dissociative symptoms, T1: 12 years of age, T2: 14 years of age | | | | | | |

| Supplementary Table 2: The trajectory of broad severe dissociative symptoms and the risk of self-harm | | | | | | |
| --- | --- | --- | --- | --- | --- | --- |
|  | broad SDIS at T1 | broad SDIS at T2 | prevalence, % | OR | 95% CI | P value |
| Trajectory of broad SDIS |  |  |  |  |  |  |
| No experience | - | - | 72.8 | 1 | reference |  |
| Incident | - | + | 8.8 | 1.74 | 0.88 to 3.42 | 0.11 |
| Transient | + | - | 7.7 | 1.25 | 0.58 to 2.69 | 0.56 |
| Persistent | + | + | 10.6 | 2.18 | 1.22 to 3.88 | <0.01 |
| Adjusted for sex and age in months. | | | | | | |
| SDIS: severe dissociative symptoms, T1: 12 years of age, T2: 14 years of age | | | | | | |
